# S1P promotes corneal trigeminal neuron differentiation and corneal nerve repair via upregulating nerve growth factor expression in a mouse model

**DOI:** 10.1515/biol-2022-0491

**Published:** 2022-10-12

**Authors:** Chaoqun Lin, Weina Li, Xuezheng Fan

**Affiliations:** Department of Neurosurgery, University of Chinese Academy of Sciences-Shenzhen Hospital (Guangming District), Shenzhen 518106, Guangdong, China; Department of Glaucoma and Cataract, Liuzhou Aier Eye Hospital, Affiliated Hospital of Aier Ophthalmology College of Central South University, 151 Liushi Road, Yufeng District, Liuzhou 545005, Guangxi, China

**Keywords:** S1P, corneal trigeminal neuron, corneal nerve, Spns2/Erk1/2 signaling pathway, nerve growth factor

## Abstract

Corneal disease was the most critical cause of vision loss. This study aimed to research a new method and provide a theoretical basis for treating corneal injury. A mice corneal epithelial injury model was constructed by the method of mechanical curettage. Models were treated with sphingosine 1-phosphate (S1P) and si-Spns2. An immunofluorescence assay was used to detect βIII-tubulin. The expressions of neurotrophic factor, S1P transporter, and extracellular signal-regulated kinase 1/2 (ERK1/2) signaling pathway-related proteins were detected by western blot. Hematoxylin–eosin staining was processed to detect the effect of SIP on corneal repair in mice. si-Spns2 inhibited the effect of S1P. S1P significantly repaired the corneal injury, while si-Spns2 treatment made it more severe. Moreover, S1P could significantly increase the levels of NGF, BDNF, GDNF, Spns2, and p-ERK1/2. si-Spns2 inhibits the effect of S1P in the expression of these proteins. S1P significantly increased axonal differentiation of trigeminal ganglion neurons, which was inhibited after si-Spns2 treatment. S1P promoted corneal trigeminal neuron differentiation and corneal nerve repair via upregulating nerve growth factor expression in a mouse model. Treatment of corneal injury by S1P may be an effective approach.

## 1 Introduction

Because the cornea is located at the front of the eyeball and plays a vital role in protecting the contents and refraction of the eye, corneal diseases have a direct impact on vision [1]. A wide variety of corneal diseases may cause loss of corneal transparency, leading to blurred vision, loss of vision, and even blindness [2]. More than 20 million people are now blinded by corneal diseases worldwide, with more than 4 million in China [3,4]. The rising incidence of corneal diseases has become the second leading cause of blindness in China [5]. Early corneal disease is curable. Keratoplasty is the primary treatment option [6]. However, in terms of global need, corneal transplantation can address only 1/70 of those in need. Corneal transplantation is not available to about 53% of the global population [7]. The lack of clinically accurate and effective treatment methods for corneal lesions is a critical problem plaguing most ophthalmologists.

The trigeminal nerve is the largest cranial nerve that originates from the pons and is divided into three branches at the trigeminal ganglion that innervates most of the facial (ophthalmic, maxillary, and mandibular), dural, and intracranial vessels [8,9]. After entering the cornea at the limbus, these nerve fibers radially pass through 1/3 of the corneal stroma, continue to bifurcate forward to form a dense subepithelial plexus, then pass forward through the anterior elastic layer, and finally reach the corneal epithelium [10]. Current studies have confirmed that corneal neuropathy is a critical cause of keratopathy [11]. Besides, neuropathy in diabetes may affect the basal inferior nerve plexus (SBP) of the cornea [12]. Axonal and dendritic growth of neuronal cell bodies is of importance in development and injury recovery [13]. The expression of silencing signal regulator (Sirt-1), calcitonin gene, and other nerve-related factors decreased in the trigeminal ganglion of the diabetic model [14].

Sphingosine 1-phosphate (S1P) is an intermediate product of lipid metabolism, which was first discovered in the brain in 1870 [15]. Until 1990, Seol et al. [16] discovered that S1P could accumulate intracellular Ca^2+^. Recent studies have shown that S1P can be used as a cell signal molecule to regulate cell proliferation, differentiation, adhesion, and migration [17]. At the same time, it can also be used as a second messenger, regulating the intracellular Ca^2+^ concentration and affecting cell growth [16]. Interestingly, evidence confirms that S1P is involved in the process of angiogenesis, including endothelial cell proliferation, survival, migration, and other physiological processes [18]. In this study, a mouse model of corneal epithelial injury was constructed to study the effect of S1P on mouse corneal trigeminal neurons and nerve repair. The aim is to find a new method and provide a theoretical basis for treating the corneal injury.

## 2 Materials and methods

### 2.1 Construction of corneal epithelial injury model and treatment

There were 21 C57 mice (adult, male, and weighing 20–25 g) with no eye examination abnormalities included in this study. All mice were purchased from the Experimental Animal Center of Nanjing University of Traditional Chinese Medicine. Among them, 18 mice received general anesthesia by intraperitoneal injection of 10% urethane solution (0.02 mL). The other three mice were used as controls. Animal experiments were divided into the control group, model group, model + S1P group or model + S1P + si-NC group, and =model + S1P + si-Spns2 group. Two subconjunctival injections (24 and 4 h) of S1P (10 μM) [19] and 5 μL si-NC (8 μM) or si-Spns2 (8 μM) were given before the injury [20], the control group and model group were injected with 0.9% normal saline in the same way. A trephine with a 2 mm diameter was used to mark the trauma area in the center of the cornea, and the corneal epithelium in the marked area was mechanically scraped.


**Ethical approval:** The research related to animal use has been complied with all the relevant national regulations and institutional policies for the care and use of animals and was approved by the experimental animal ethics committee of the University of Chinese Academy of Sciences-Shenzhen Hospital (Guangming District) (LL-KT-2020111).

### 2.2 Acute isolation of trigeminal ganglion cells and treatment

After the injury model was established for 24 h, the mice were sacrificed by dislocation after anesthesia. The trigeminal ganglia on both sides were taken and placed in dulbecco's modified eagle medium (DMEM)/F12 medium (Gibco, supplied with 14 mM/L NaHCO_3_, pH = 7.4) for washing and incubation. After the sample was cut into pieces with iris scissors, the sample was incubated for 13 min in a constant temperature water bath shaker (36°C, 140 rpm) by enzymatic digestion (0.2 g/L collagenases I, sigma; 0.1 g/L trypsin III, sigma) and gently pipetted several times with a heat-treated Pasteur pipette. Finally, 0.3 g/L trypsin inhibitor was added to terminate the digestion. The cells were filtered through a 200-mesh nylon sieve into a 35 mm culture dish, and the outer fluid was replaced twice after the cells adhered to the wall.

To study the effect of S1P on the axon differentiation of trigeminal ganglion neurons, the cells were divided into the S1P (Enzo Biochem) group and the phosphate buffered solution (PBS) group. For the S1P group, the added 2.0 μM S1P was cultured for 6 h [21]. PBS-treated cells at the same dose served as controls.

To study the role of protein spinster homolog 2 (Spns2) in the axon differentiation of trigeminal ganglion neurons induced by S1P, the cells were divided into three groups (PBS group, S1P or S1P + si-NC group, and S1P + si-Spns2 group). si-Spns2 and corresponding control were designed and synthesized by GL Biochem Ltd. (Shanghai, China). A total of 50 pmol of si-Spns2 or si-NC group was added to the serum-free DMEM and mixed thoroughly to prepare the RNA diluent with a final volume of 25 μL. One microliter of Entranster-R4000 was added to 24 µL of serum-free DMEM liquid and mixed well to make Entranster-R4000 dilution, and the final volume was 25 μL. Entranster-R4000 diluent and RNA diluent were mixed thoroughly and stayed at room temperature for 15 min. Then, 50 μL of transfection complex was added dropwise to the cells with 0.45 mL of complete medium and mixed well. After transfection for 6 h, the cell morphology changed was observed under the light microscopy (Leitz, Germany), the medium was changed, and the culture was continued for 48 h. For the S1P + si-NC group and S1P + si-Spns2 group, 2.0 μM S1P was added when siRNA was transfected.

### 2.3 Hematoxylin and eosin (HE) staining

The corneal tissues of mice in each group were obtained and placed in 40 g/L of paraformaldehyde solution for tissue fixation. After dehydration with ethanol, the tissues were immersed in paraffin and embedded, sectioned routinely, and stained with HE. Under an optical microscope, the changes in mouse corneal epithelial structure were observed and photographed. And epithelial thickness was measured from the top of the corneal epithelium to the basement membrane using ImageJ (ImageJ 1.51j8, National Institutes of Health, USA). Three central measurements were taken from each section (lateral interval 50 μm) and averaged to obtain thickness values for each cornea [22].

### 2.4 Immunofluorescence detection

βⅢ-tubulin is a highly dynamic tubulin isotype mainly expressed in developing neurons and is often used as a marker protein for neurons [23,24].

Fetal bovine serum (10%) treatment was set as the control group, and the experimental group was the S1P group with 2.0 μM/L, and each group was repeated three times. The cultured cells were seeded in a 48-well plate at a density of 1 × 10^5^ cells per well and cultured at 37°C for 24 h and then washed with phosphate-buffered saline and fixed with 4% paraformaldehyde (Sigma Aldrich) for 10 min at 4°C. Cells were again rinsed with phosphate-buffered saline and permeabilized with 1% Triton X-100 for 10 min at 4°C. Subsequently, cells were incubated with βⅢ-tubulin antibodies (ab7291) overnight at 37°C. Then, goat anti-rabbit IgG H&L secondary antibodies (ab150077) were added for 1 h incubation.

After the corneas were cut longitudinally into standard sections, they were incubated in 20 mmol/L EDTA for 30 min at 37°C, followed by 2 days of incubation in 0.025% hyaluronidase and 0.1% EDTA in PBS. Tissues were blocked in PBS Triton X-100 (containing 2% albumin from bovine serum) for 2 h at room temperature and then incubated overnight at 4°C with βⅢ-tubulin antibodies (ab7291). Then, goat anti-rabbit IgG H&L secondary antibody (ab150077) were incubated overnight.

Under the fluorescence microscope, the image analysis software Motic Digital Class 1.1/Motic Images Advanced 3.1 was used to measure the processes of neurons. The mean fluorescence intensity of each group was analyzed using ImageJ (ImageJ 1.51j8).

### 2.5 Western blot analysis

A total of 1 mL of RNA immunoprecipitation lysis buffer and 10 μL of phenylmethanesulfonyl fluoride were used to decompose and differentiate cells and tissues in each group. After the cells were sonicated and centrifuged, the supernatant was added with protein-loading buffer and treated at 100°C for 10 min. A total of 10 g of sample was loaded on a 10% sodium dodecyl sulfate polyacrylamide gel electrophoresis gel for electrophoresis. After the electrophoretic separation, the protein was transferred to the PVDF membrane for 90 min. The sample was blocked with 5% skimmed milk powder for 60 min, and the primary antibody (NGF, ab52918; BDNF, ab108319; GDNF, ab176564; Spns2, ab82629; p-Erk1/2, ab201015; Erk1/2, ab115799; Abcam) was added to incubate for 2 h at room temperature. After washing, the horseradish peroxidase secondary antibody (ab6721) was added and placed at 4°C overnight. The membrane was washed three times by tris-buffered saline with Tween-20 and exposed in the ECL chemiluminescence kit (Pharmacia-Amersham, Piscataway, NJ, USA) darkroom. ImageJ software performed grayscale analysis. β-Actin was used as an internal reference to calculate the expression of each protein.

### 2.6 Statistical analysis

All experimental data were obtained from three independent repeated experiments. The experimental data were expressed as mean ± standard deviation, and SPSS 22.0 statistical software was used to analyze the data. One-way analysis of variance was used for comparison among multiple groups, and the Dunnett *t*-test or least significant difference (LSD) *t*-test was used for further comparison between groups. *P* < 0.05 represented statistical significance.

## 3 Results

### 3.1 S1P promoted the axon differentiation of trigeminal ganglion neurons

**Figure 1:**
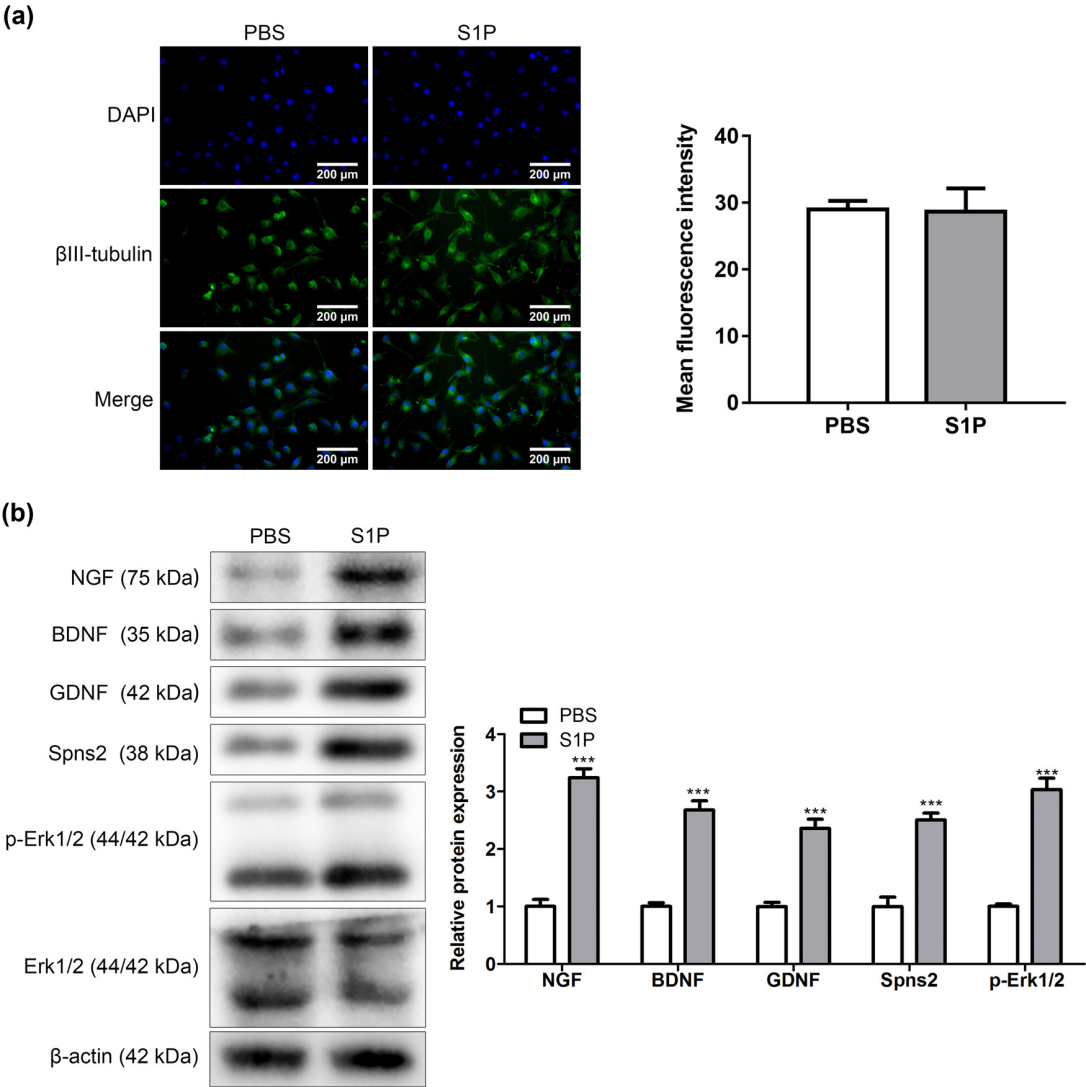
S1P promoted the axon differentiation of trigeminal ganglion neurons. (a) Immunofluorescence detection of βIII-tubulin (*n* = 3). (b) Expression of neurotrophic factor, sphingosine-1-phosphate transporter, and ERK1/2 signaling pathway-related proteins (*n* = 3). ^***^ *P <* 0.001. The Dunnett *t*-test or LSD *t*-test was used for further comparison between groups.

To study the effect of S1P on the axon differentiation of mouse trigeminal ganglion neurons, the experiment was divided into S1P and PBS groups. The immunofluorescence method was used to detect the expression of βIII-tubulin. After S1P treatment, axonal differentiation of mouse trigeminal ganglion neurons was significantly increased. However, the mean fluorescence intensity of each group was analyzed using ImageJ and found no significant difference between the two groups (Figure 1a). Western blot assay was undertaken to detect neurotrophic factors, S1P transporter, and ERK1/2 signaling pathway-related proteins. As a result, the expressions of NGF, BDNF, GDNF, Spns2, and p-Erk1/2 were significantly higher in S1P groups than in PBS groups. However, Erk1/2 was no significant difference between the two groups (Figure 1b). Thereby, S1P promoted the axon differentiation of trigeminal ganglion neurons.

### 3.2 si-Spns2 inhibited the axon differentiation of trigeminal ganglion neurons

**Figure 2:**
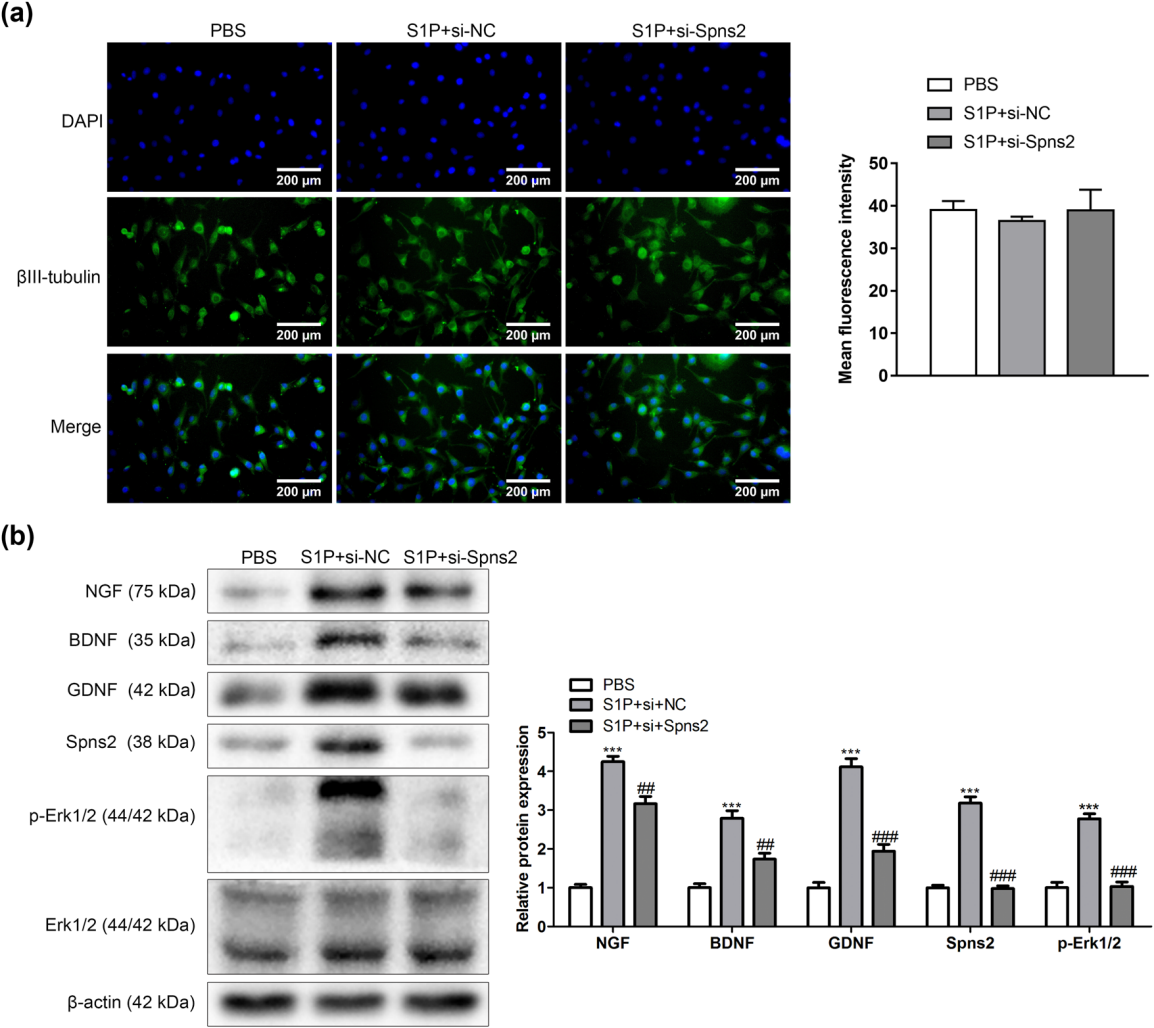
si-Spns2 inhibited the axon differentiation of trigeminal ganglion neurons. (a) Immunofluorescence detection of βIII-tubulin (*n* = 3). (b) Expression of neurotrophic factor, sphingosine-1-phosphate transporter, and ERK1/2 signaling pathway-related proteins (*n* = 3). Compared with the PBS group, ^***^ *P* < 0.001. Compared with S1P + si + NG group, ^##^ *P* < 0.01, ^###^ *P* < 0.001. One-way analysis of variance was used for comparison among multiple groups.

To research the effect in the axon differentiation of trigeminal ganglion neurons induced by S1P, the cells were divided into the PBS group, S1P + si-NC group, and S1P + si-Spns2 group. S1P significantly increased axonal differentiation of trigeminal ganglion neurons and also promoted the expression of neurotrophic factors, S1P transporter, and ERK1/2 signaling pathway-related proteins. Interestingly, si-Spns2 inhibits the effect of S1P (Figure 2a and b).

### 3.3 SIP repaired the cornea of a mouse model

**Figure 3:**
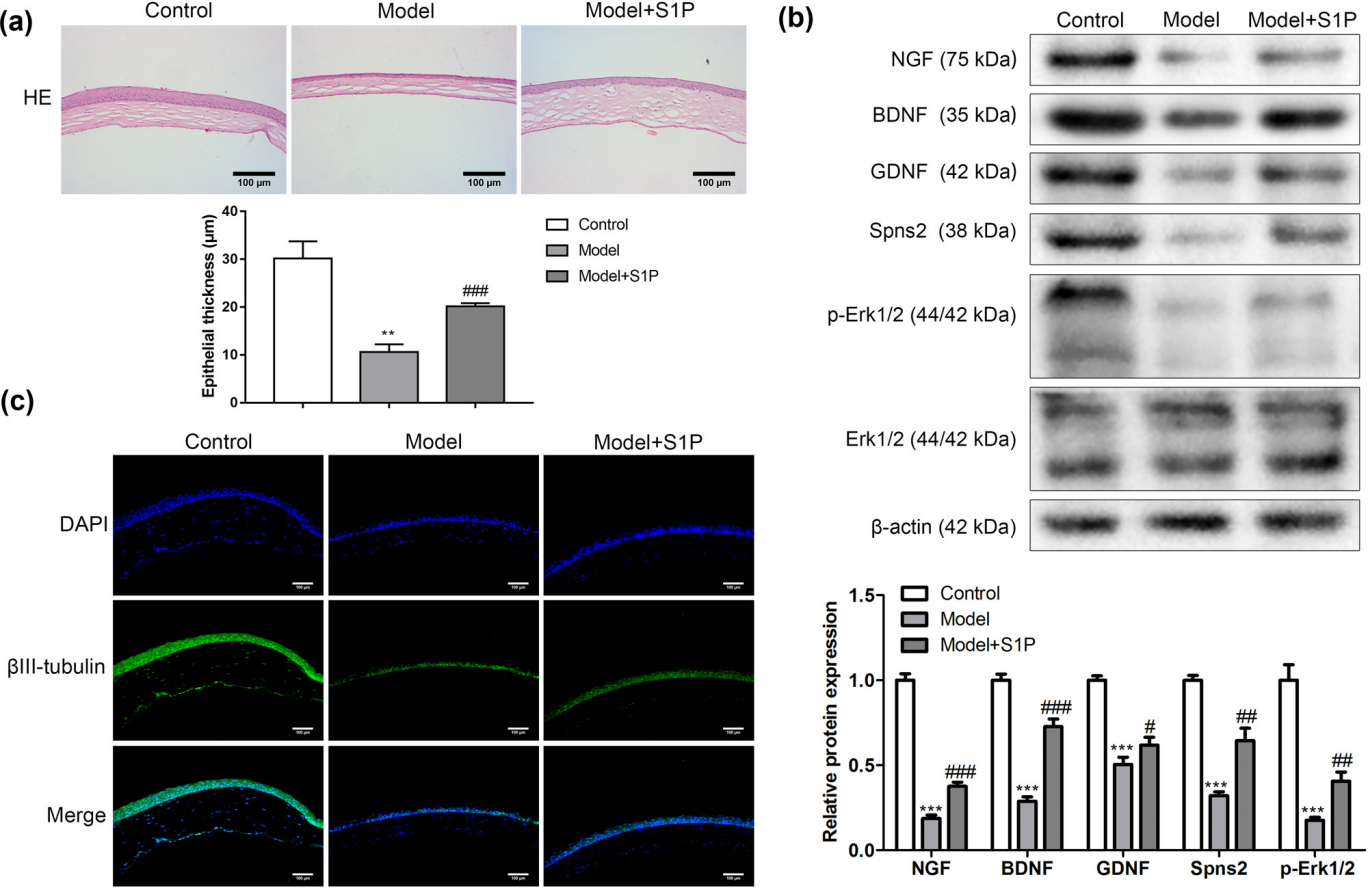
SIP repaired the cornea of the mouse model. Based on the treatment, the cornea samples of the mouse were divided into three groups, including control, model, and model + S1P groups. (a) The corneal (longitudinal section of cornea) damage was detected by HE staining and the corneal epithelial thickness was measured with ImageJ (*n* = 3). (b) Western blot assay was processed to detect neurotrophic factors, sphingosine 1-phosphate transporter, and ERK1/2 signaling pathway-related proteins (*n* = 3). (c) Immunofluorescence detection for βIII-tubulin (*n* = 3). Compared with the control group, ^***^ *P <* 0.001. Compared with model group, ^#^ *P <* 0.05, ^##^ *P <* 0.01, ^###^ *P <* 0.001. One-way analysis of variance was used for comparison among multiple groups.

Based on the treatment, mouses were divided into three groups, including control, model, and model + S1P groups. In the model group, the corneal damage of the mice was severe, and after S1P treatment, the corneal damage was alleviated and the corneal epithelial thickness was significantly increased, but it was still more severe than in the control group (Figure 3a). Western blot assay was processed to detect neurotrophic factors, S1P transporter, and ERK1/2 signaling pathway-related proteins. As shown in Figure 3b, the expressions of NGF, BDNF, GDNF, Spns2, and p-Erk1/2 were significantly lower in model groups than in control. Interestingly, S1P treatment significantly increased these protein levels. On the other hand, Erk1/2 was no significant difference among the three groups (Figure 3b). Similarly, βIII-tubulin immunofluorescence assay results showed that neuronal axonal differentiation of trigeminal ganglion was obviously decreased in the model group, while S1P treatment could restore the axonal differentiation of trigeminal ganglion neurons (Figure 3c).

### 3.4 Spns2 activated Spns2/Erk1/2 signaling pathway and upregulated nerve growth factors

**Figure 4:**
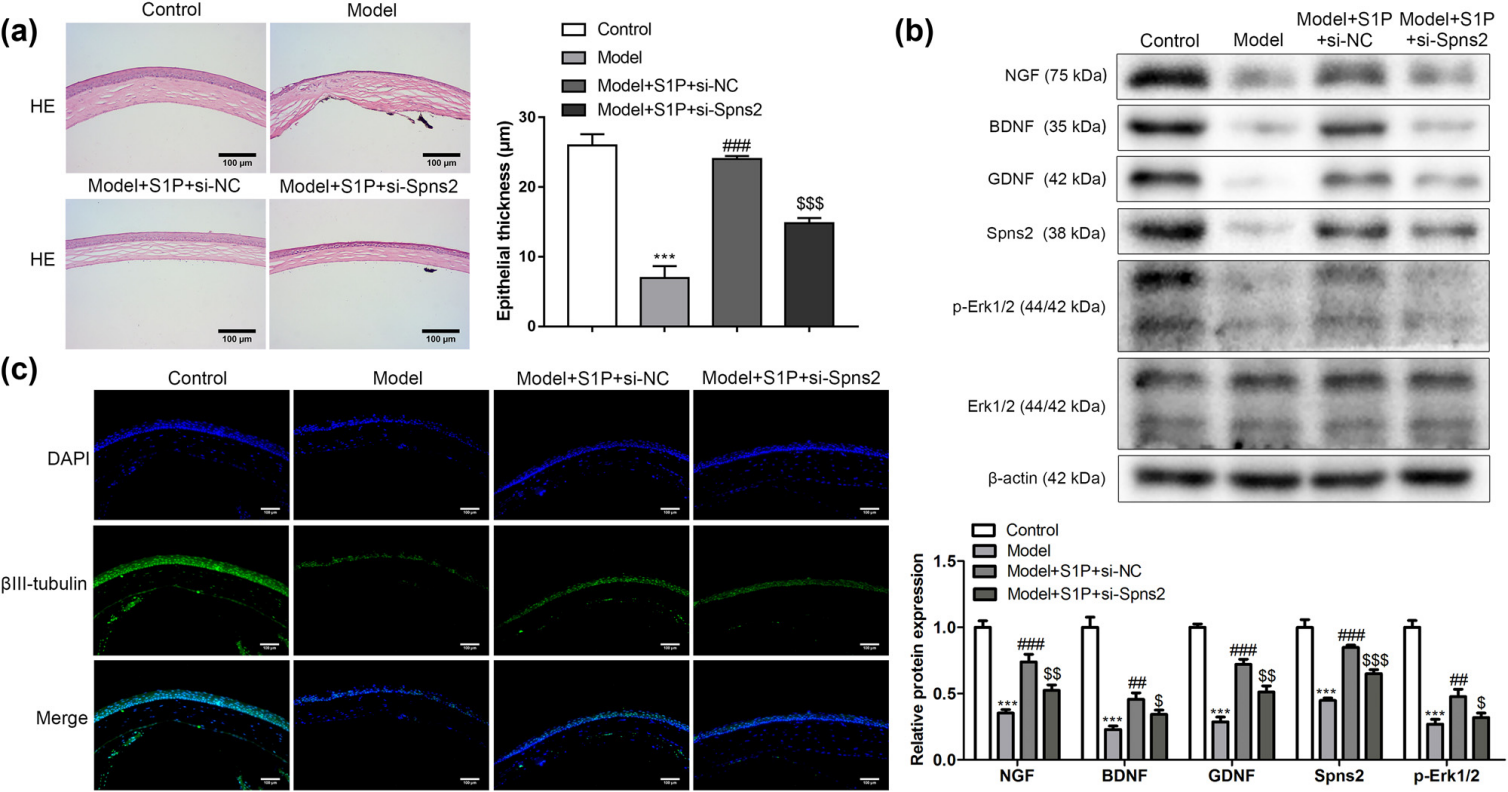
Spns2 activated Spns2/Erk1/2 signaling pathway and upregulated nerve growth factors. To research the role of Spns2 in the cornea repairing process, the cornea samples of mice were divided into four groups, including control, model, model + S1P + si-NC, and model + S1P + si-Spns2 groups. (a) S1P significantly repaired the corneal (longitudinal section of cornea) injury compared with the control group, while si-Spns2 treatment made it more severe (*n* = 3). (b) si-Spns2 reversed the effect of S1P in the expressions of NGF, BDNF, GDNF, Spns2, and p-Erk1/2 (*n* = 3). (c) Axonal differentiation of trigeminal ganglion neurons increased significantly in model + S1P + si-NC groups than in model control, while si-Spns2 treatment inhibited the function (*n* = 3). Compared with the control group, ^***^ *P <* 0.001. Compared with model group, ^##^ *P <* 0.01, ^###^ *P <* 0.001. Compared with model + S1P + si-NC group, ^$^ *P <* 0.05, ^$$^ *P <* 0.01, ^$$$^ *P <* 0.001. One-way analysis of variance was used for comparison among multiple groups.

To research the role of Spns2 in the cornea repairing process, the cornea samples of the mouse were divided into four groups, including control, model, model + S1P + si-NC, and model + S1P + si-Spns2 groups. S1P significantly repaired the corneal injury and increased the corneal epithelial thickness compared with the control group, while si-Spns2 treatment made it more severe (Figure 4a). Moreover, the levels of NGF, BDNF, GDNF, Spns2, and p-Erk1/2 were significantly higher in model + S1P + si-NC groups than those in model control. It was worth noting that si-Spns2 inhibited the effect of S1P in the expression of these proteins (Figure 4b). Furthermore, axonal differentiation of trigeminal ganglion neurons increased significantly in model + S1P + si-NC groups than those in model control, while si-Spns2 treatment inhibited the function. Based on this evidence, Spns2 activated Spns2/Erk1/2 signaling pathway and upregulated nerve growth factors.

## 4 Discussion

The clinical research on corneal disease has made significant progress, but many problems remain [25]. Although molecular biology techniques have been introduced into the study of corneal diseases, the pathogenesis of many corneal diseases has not yet been elucidated. Furthermore, genetic treatment is still at the research level of the laboratory, and there is still a long way to go to the clinical application [26]. Thereby, it is of great significance to study the mechanism of the effective drug during corneal disease treatment. In this study, a corneal epithelial injury model was constructed. Excitingly, the results in this study showed that SIP had a repairing effect on the cornea of a mouse model. Moreover, S1P promoted the axon differentiation of trigeminal ganglion neurons, which caught our attention.

S1P is a metabolite of plasma membrane sphingomyelin [27]. It can be used as an extracellular ligand to bind to the corresponding receptor (S1PR) to induce the activation or inhibition of various signal pathways, affect many essential cell processes, and trigger various biological effects [28]. In a previous study, an S1PR inhibitor successfully reduced immunosuppression and inflammation during corneal transplantation [29]. Moreover, during the treatment of ocular uveitis, its application effectively reduces the accumulation of CD^4+^ and IL-17^+^ T cells in the ocular inflammation site [30,31]. Thereby, the effect and mechanism of S1P receptor inhibitors in ocular diseases is an essential direction of ophthalmology research. Besides, Quintyn et al. [32] referred that intravitreal injection of S1P significantly reduced the damage of photoreceptor cells caused by retinal detachment. In addition, Miranda et al. confirmed *in vitro* experiments that S1P attenuated the oxidative stress response of photoreceptor cells, thereby effectively avoiding the occurrence of cell apoptosis [33].

Furthermore, the results in this study confirmed that SIP accelerated the expression of Spns2. Spns2 is a membrane transport protein discovered in recent years, which can specifically mediate the secretion of S1P in the body [34]. Previous studies have confirmed that the damage to the retinal structure of Spins2 mutant rats during early birth was caused by the loss of the Spns transporter [35,36]. Interestingly, si-Spns2 inhibited the axon differentiation of trigeminal ganglion neurons in this study. Thereby, S1P and Spns2 promoted corneal trigeminal neuron differentiation and corneal nerve repair.

It was worth noting that S1P and Spns2 activated Spns2/Erk1/2 signaling pathway in this study. In previous studies, the ERK signaling pathway was activated in diabetic retinopathy, and the activity of the ERK signaling pathway increased with the development of the disease, suggesting that the activation of the RAS system in diabetic retinopathy was related to the ERK signaling pathway [37]. In addition, the ERK1/2 signaling pathway was confirmed to regulate the proliferation of corneal stromal cells and play a critical role in corneal stromal cell damage [38]. Niu et al. confirmed that *Aspergillus fumigatus* increased the expression of PAR-2 in the cornea and upregulated the expression of proinflammatory cytokines through the PAR-2/ERK1/2 signaling pathway [39]. Similar results were also obtained in this study. The levels of Spns2 and p-Erk1/2 were significantly higher in model + S1P + si-NC groups than those in model control. si-Spns2 inhibited the effect of S1P in the expression of these proteins.

Furthermore, S1P and Spns2 upregulated nerve growth factors in this study. Thereinto, NGF, BDNF, GDNF, Spns2, and p-Erk1/2 were significantly higher in S1P treatment groups. Early studies have shown that there were endogenous NGF and its receptors in the iris, lens, retina, and optic nerve of animals [40]. After severing the axons of retinal ganglion cells (RGC), providing NGF prevented RGC from death [41]. Okada et al. referred that NGF promoted the mitosis of corneal epithelial cells and limbal stem cells cultured *in vitro* and accelerated their growth and differentiation [42]. Significantly, NGF promoted the proliferation and migration of corneal epithelial cells by increasing the expression of gene metalloproteinase-9 and TrkA [43]. In addition, BDNF played a critical role in protecting and prolonging the survival of retinal ganglion cells after optic nerve injury, regenerating optic nerve axons, and protecting photoreceptor cells [44]. More importantly, the connection of BDNF, CNTF, FGF, and NGF caused the activation of intercellular signal linkages to have a significant overlap [45].

In conclusion, S1P promoted the differentiation of corneal trigeminal neurons and upregulated nerve growth factor by activating the Spns2/Erk1/2 signaling pathway to promote corneal nerve repair in a mouse model. Thus, the study may provide a new therapy method for corneal nerve repairing.

To more comprehensively investigate the role and mechanism of S1P in the treatment of corneal injury, studies still need to be conducted from more aspects, such as the interaction with other proteins or signaling pathways on the mechanism of corneal injury. In addition, a nerve cell injury model can be constructed by lipopolysaccharide to investigate the mechanism of S1P on nerve injury repair from the cellular level.

## Supplementary Material

Supplementary Figure
