# Supplementary material for: S1P promotes corneal trigeminal neuron differentiation and corneal nerve repair via upregulating nerve growth factor expression in a mouse model
Source: Open Life Sci. 2022 Oct 12;17(1):1324–32. doi: 10.1515/biol-2022-0491 (PMC9559473; doi:10.1515/biol-2022-0491)
Supplement: Supplementary Figure [file biol-2022-0491-sm.pdf]

Supplementary materials

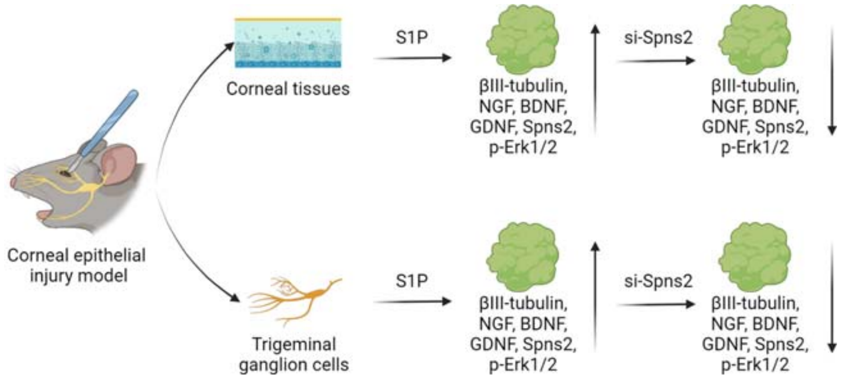

Figure S1: Mechanism map of S1P promoting corneal trigeminal nerve differentiation and corneal nerve repair by upregulating NGF expression.
